# Too much data, but little inter-changeability: a lesson learned from mining public data on tissue specificity of gene expression

**DOI:** 10.1186/1745-6150-1-33

**Published:** 2006-10-25

**Authors:** Shuyu Li, Yiqun Helen Li, Tao Wei, Eric Wen Su, Kevin Duffin, Birong Liao

**Affiliations:** 1 Integrative Biology, Lilly Research Laboratories, Eli Lilly and Company, Lilly Corporate Center, Indianapolis, IN 46285, USA; 2 Discovery Informatics, Lilly Research Laboratories, Eli Lilly and Company, Lilly Corporate Center, Indianapolis, IN 46285, USA

## Abstract

**Background:**

The tissue expression pattern of a gene often provides an important clue to its potential role in a biological process. A vast amount of gene expression data have been and are being accumulated in public repository through different technology platforms. However, exploitations of these rich data sources remain limited in part due to issues of technology standardization. Our objective is to test the data comparability between SAGE and microarray technologies, through examining the expression pattern of genes under normal physiological states across variety of tissues.

**Results:**

There are 42–54% of genes showing significant correlations in tissue expression patterns between SAGE and GeneChip, with 30–40% of genes whose expression patterns are positively correlated and 10–15% of genes whose expression patterns are negatively correlated at a statistically significant level (p = 0.05). Our analysis suggests that the discrepancy on the expression patterns derived from technology platforms is not likely from the heterogeneity of tissues used in these technologies, or other spurious correlations resulting from microarray probe design, abundance of genes, or gene function. The discrepancy can be partially explained by errors in the original assignment of SAGE tags to genes due to the evolution of sequence databases. In addition, sequence analysis has indicated that many SAGE tags and Affymetrix array probe sets are mapped to different splice variants or different sequence regions although they represent the same gene, which also contributes to the observed discrepancies between SAGE and array expression data.

**Conclusion:**

To our knowledge, this is the first report attempting to mine gene expression patterns across tissues using public data from different technology platforms. Unlike previous similar studies that only demonstrated the discrepancies between the two gene expression platforms, we carried out in-depth analysis to further investigate the cause for such discrepancies. Our study shows that the exploitation of rich public expression resource requires extensive knowledge about the technologies, and experiment. Informatic methodologies for better interoperability among platforms still remain a gap. One of the areas that can be improved practically is the accurate sequence mapping of SAGE tags and array probes to full-length genes.

**Reviewers:**

This article was reviewed by Dr. I. King Jordan, Dr. Joel Bader, and Dr. Arcady Mushegian.

## Open peer review

Reviewed by Dr. I. King Jordan, Dr. Joel Bader, and Dr. Arcady Mushegian. For the full reviews, please go to the Reviewers' comments section.

## Background

The advent of high throughput molecular technologies has led to a data explosion in biology. One of the gene expression repositories, Gene Expression Omnibus, contains approximately half a billion measurements on individual genes of more than 100 organisms, from 30,000 submissions in the fall of 2004 [1]. The public gene expression data is expected to increase exponentially with the requirement of mandatory submission by majority of scientific journals [2]. These databases remain a treasure trove of information that can be mined to enhance research productivity and quality (new targets, biomarkers, etc.). For example, Mootha et al. (2003) was able to identify human disease-causing genes through integration of data from different species [3]. However, to date, the integration/exploitation of these rich data sources and translation to information and knowledge is grossly limited in part due to the lack of standardization across different platforms used for generating the deposited data. The exploitation of this rich resource remains quite tedious and arduous even for the competent "power users". Careful comparisons across platforms are needed to assess data comparability and to increase confidence in the utility and interpretation of the results.

Two of the most widely used platforms for generating gene expression data are serial analyses of gene expression (SAGE) and Affymetrix GeneChip^® ^[4,5]. GeneChip or microarray platform has dominated the expression market in the past several years. However, the SAGE technology is gradually gaining popularity for its capability in unbiased identification of novel transcripts for gene discovery. Literature reports have shown conflict results when direct comparisons of quantitative data between the two platforms were made on individual cell lines, with one report showing good correlations [6] and another showing poor to moderate correlations between these two platforms [7]. Van Ruissen et al [8] has also designed mathematically a new method for comparison, namely between-ratio difference as a concordance measure per gene, and concluded that the overall concordance between these two platforms was modest.

The aim of this project is to test the data interchangeability/comparability of tissue expression pattern of gene using public data generated by different technology platforms, rather than direct technology comparisons as in [6,7], for several reasons. First, biologists are expected in the future to leverage the vast amount of public expression data more rather than generating their own data, especially data from well annotated tissues with regard to its physiological states (disease, normal, etc.) Secondly, tissue-specific expression pattern is an important factor for deciding if a gene is a suitable target for drug intervention, or suitable as a potential biomarker for drug activity/efficacy or disease progression. In this report, we computed the tissue distribution pattern for each gene using data generated from tissues under normal physiological states with either SAGE or microarray technology, and then their gene expression patterns were compared between SAGE and microarray platforms. We found the big discrepancies on the tissue expression pattern of genes in different platforms and identified several factors that may contribute to discrepancies. Recommendations were made to better mine public expression data.

## Results

### Tissue expression pattern of genes between SAGE and microarray platforms differed

Tissue distribution of gene expression is important in understanding gene function and regulation, and it provides biologists some clues if a gene is druggable or can be a potential biomarker. We set out to compare the tissue distribution of gene expression across two major platforms, SAGE and microarray, where the high throughput data were recently obtained. We first identified the datasets generated with tissue samples from normal physiological states using the SAGE technology, and then examined the datasets annotated with the same tissue type of normal physiological states, but generated with Affymetrix microarray platforms (Table 1). Thirteen tissues were identified to have data available from both platforms. Affymetrix probe sets and SAGE tags were mapped to Locuslink IDs based on NetAffx and NCBI annotations, respectively. A total of 7536 loci are common between the SAGE and array datasets. Due to differential gene expression and the depth of tag sequencing in SAGE experiments, some genes do not have a tag count data in a particular experiment. Table 2 illustrates the distributions of gene loci with respect to the number of tissues that have tag counts available. Only a small number of genes have tag count data in all tissues.

**Table 1:** Tissue Mapping Between Datasets

**Tissue**	**Array (GDS422)**	**Array (GDS596)**	**Array (GDS181)**	**SAGE**
Brain	Brain_17	WholeBrain_1	Wholebrain_1	WholeBrain_GSM763
Brain	Brain_18	WholeBrain_2	Wholebrain_2	WholeBrain_GSM676
Cerebellum		Cerebellum_1	Cerebellum_1	Cerebellum_GSM761
Cerebellum		Cerebellum_2	Cerebellum_2	Cerebellum_GSM695
FrontalCortex		FrontalCortex_1		FrontalCortex_GSM786
FrontalCortex		FrontalCortex_2		
Heart	Heart_5	Heart_1	Heart_1	Heart_GSM1499
Heart	Heart_6	Heart_2	Heart_2	
Kidney	Kidney_11	Kidney_1	Kidney_1	Kidney_GSM708
Kidney	Kidney_12	Kidney_2	Kidney_2	
Kidney			Kidney_3	
Liver	Liver_3	Liver_1	Liver_1	Liver_GSM785
Liver	Liver_4	Liver_2	Liver_2	
Lung	Lung_10	Lung_1	Lung_1	Lung_GSM762
Lung	Lung_9	Lung_2	Lung_2	
Ovary		Ovary_1	Ovary_epithelium_1	Ovary_GSM719
Ovary		Ovary_2		
Pancreas	Pancreas_23	Pancreas_1	Pancreas_1	Pancreas_GSM721
Pancreas	Pancreas_24	Pancreas_2	Pancreas_2	Pancreas_GSM716
Placenta		PLACENTA_1	Placenta_1	Placenta_GSM14750
Placenta		PLACENTA_2	Placenta_2	Placenta_GSM14749
Prostate	Prostate_21	Prostate_1	Prostate_1	Prostate_GSM764
Prostate	Prostate_22	Prostate_2	Prostate_2	Prostate_GSM685
Prostate			Prostate_3	Prostate_GSM739
Prostate				Prostate_GSM14752
SpinalCord	Spinal_cord_19	SpinalCord_1	Spinal_cord_1	SpinalCord_GSM2386
SpinalCord	Spinal_cord_20	SpinalCord_2	Spinal_cord_2	
Thalamus		Thalamus_1	Thalamus_1	Thalamus_GSM713
Thalamus		Thalamus_2	Thalamus_2	

**Table 2:** Distribution of gene numbers and tissue types with available SAGE tag counts

Tissue types	The number of locus entries
13	20
12	31
11	20
10	32
9	51
8	36
7	50
6	71
5	498
4	708
3	1019
2	1570
1	5363

**Figure 1:**
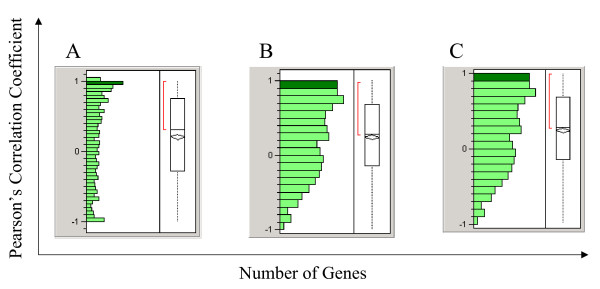
Distribution of correlation coefficient in tissue distribution between SAGE and microarray platforms. (A) Correlation between SAGE and GDS422; (B) Correlation between SAGE and GDS181; (3) Correlation between SAGE and GDS596. X-axis represents the number of genes in each correlation category; Y-axis represents the correlation coefficient. There were similar numbers of negative correlation as positive correlation, with slightly more positive correlated genes between the platforms.

There were 8 common tissue types between SAGE and GDS422, 12 between SAGE and GDS181, and 13 between SAGE and GDS596 data sets. Correlation coefficients were calculated for genes having SAGE tag count data in at least 3 common tissues between SAGE and GDS422, or at least 5 common tissues between SAGE and GDS181 or between SAGE and GDS596. Figure 1 revealed the distribution of correlation coefficients of tissue expression pattern between SAGE and microarray data sets. There are significantly more positive correlations than negative correlations. The statistical significance of correlation was determined by t-tests using p value of 0.05 as the threshold. Table 3 showed the breakdown of statistically significant correlations between SAGE and microarray data sets. Of significant correlations, ranging from 42 to 54%, there were at least three times more positively correlated expression patterns (31.7 – 38.8%) than negatively correlated ones (9.2 – 15.3%).

**Table 3:** Breakdown of correlation between SAGE and Microarray

Categories	GDS596	GDS181	GDS422
Total Entries^1^	1618	1091	1041
Significant	684 (42.3%)	512 (46.9%)	563 (54.1%)
Positive	513 (31.7%)	412 (37.8%)	404 (38.8%)
Negative	171 (10.6%)	100 (9.2%)	159 (15.3%)

^1 ^total number of loci whose expression were measured by both SAGE and microarray in at least 3 or 5 tissues.

### The difference in tissue expression pattern of genes between platforms was not related to the origins of tissue samples

We decided to trace down the causes of no or negative correlations in tissue expression patterns between the two platforms. Since SAGE and microarray profiling used different biological samples, which were considered the biggest source of variation in gene expression studies, correlations between SAGE and array expression data for each tissue were calculated and summarized in Table 4. The correlations varied greatly among the tissues. However, with the exception of ovary samples, the distribution of correlation coefficients were similar to those (0.29–0.51) reported by Lu et al. (2004) when the same samples were used. In several cases, correlations were even higher than what was reported by Lu et al. We also measured correlations by Spearman rank correlation analysis and obtained similar results (data not shown).

**Table 4:** Correlation between SAGE and Microarray by Tissue Type

	*GDS596*	*GDS422*	*GDS181*
Heart	0.40	0.61	0.30
Liver	0.34	0.61	0.38
Lung	0.32	0.43	0.48
Kidney	0.24	0.23	0.34
Prostate	0.31	0.47	0.66
Frontal Cortex	0.49		
Spinal Cord	0.32	0.23	0.51
Cerebellum	0.40		0.53
Thalamus	0.39		0.47
Placenta	0.34		0.48
Whole Brain	0.25	0.71	0.31
Pancreas	0.29	0.32	0.33
Ovary	0.02		0.16

### The difference in tissue gene expression pattern between platforms is not related to number of common tissues between the platforms, gene function, gene abundance and design of array probe sets

**Figure 2:**
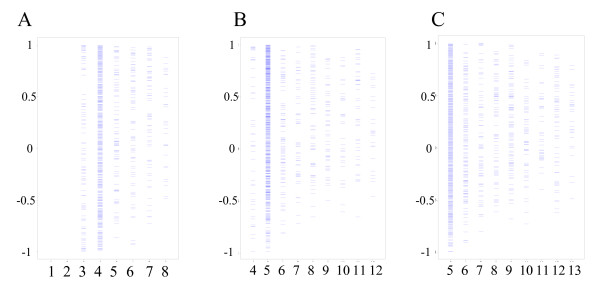
The distribution of correlation coefficients across the number of common tissue types that data are available for both microarray and SAGE platforms. (A)SAGE and GDS422; (B) SAGE and GDS181; (C) SAGE and GDS596. X-axis represents the number of common tissue types, Y-axis represents the correlation coefficient.

Due to various depth of sequencing in SAGE experiments, each SAGE tag only has sequence count data in a subset of tissues. Therefore, the number of common tissues with available expression data between two platforms is different for different genes. We wanted to test if there were spurious correlations in genes with small numbers of common tissues, scatter plots were drawn to explore this possibility. Figure 2 showed the distribution of correlation coefficients across the number of common tissue types between these two platforms, there was no obvious relationship between them. Similar results were observed between the significant correlations and the number of common tissues (data not shown). Therefore, it is unlikely that number of common tissues between platforms contributes the difference in tissue expression pattern.

Attempts were made to examine if particular classes of genes showed consistent correlations (either positive or lack of). We classified genes into functional groups and found that there were no obvious gene groups of particular functional categories that showed consistent correlations between SAGE and GDS596. Genes such as transcription factors, metabolism genes, housekeeping genes, etc were in both positively and negatively correlated groups (data not show). Same results were found between SAGE and GDS181 or between SAGE and GDS422 data sets. For some gene loci, more than one Affymetrix probe sets or SAGE tags have been mapped to the same locus. We observed that within a given locus, there were Affymetrix probe sets that were either positively or negatively correlated with that of SAGE tags [see Additional file 1]. Probe set annotations indicated that these gene loci exhibiting inconsistent correlations did not fall into particular functional class.

**Figure 3:**
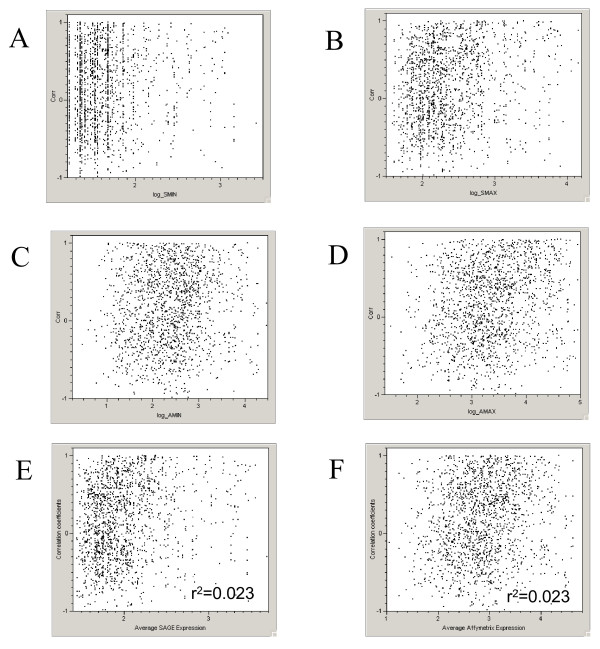
The distribution of correlation coefficients across the expression level of genes in SAGE and microarray platforms. X-axis represents the correlation coefficient and Y-axis represents the expression level. (A, B, E) Low, high and average expression levels across the significant correlation from GDS596. (C, D, F) Low, high and average expression levels across the significant correlation from SAGE data. Expression values were log10 transformed.

It has been reported that there are substantially more variations in expression measurements for genes with low abundance than that of highly expressed genes. We investigated if high abundance genes were more likely than low abundance genes to have positive correlations between SAGE and array expression data. Figure 3 illustrated that correlation coefficients distributed evenly across the lowest and highest expression value for a given gene in both GDS596 (A) and SAGE (B). Therefore, gene expression level and number of SAGE count were unlikely to contribute to the differences in tissue expression pattern between the platforms.

**Figure 4:**
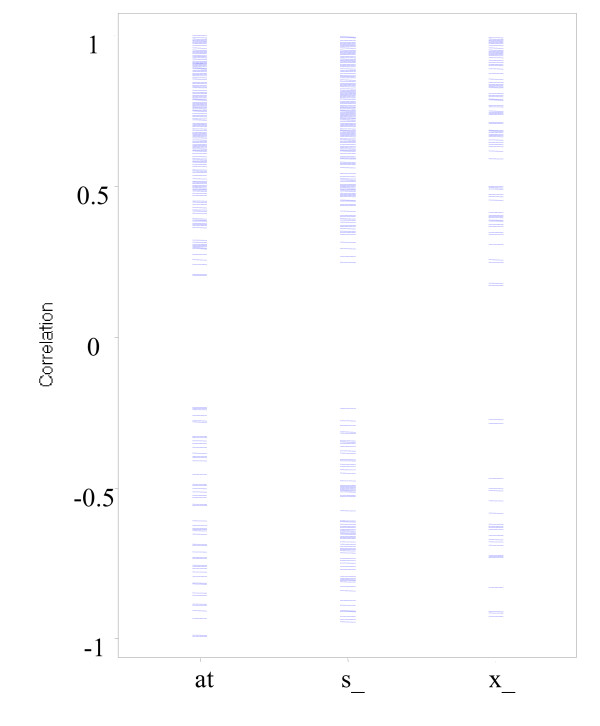
The distribution of correction coefficient across design platform of Affymetrix probe sets. Legend: "at" represents probe sets appended with "_at", for reliable probe sets. "s_" represents probe sets appended with "_s_at" for probes that may hybridize to multiple transcripts. "X_" represents "_x_at" for probes that may hybridize to unrelated transcripts.

It has been widely recognized that the hybridization between DNA and RNA depends on the composition of probing sequences and specificity of sequence to a given gene. Due to the similarity of DNA sequences and difficulty of designing specific probes for every gene in the human genome, Affymetrix has designated different types of probe set names appended with "_at", "_s_at" or "_x_at" [9] to represent the probe set sequence specificity. A simple "_at" extension in probe set IDs represents unique probe set sequence. A probe set name is appended with the "_s_at" extension if probes match multiple transcripts of the same gene or transcripts of homologous genes. Those probe sets appended with the "_x_at" extension contain some probes that are identical, or highly similar, to unrelated sequences and may cross-hybridize in an unpredictable manner with sequences other than the main target. Figure 4 illustrated the distribution of correction coefficients across various designs of Affymetrix probe sets. There was no obvious difference among the probe designs in terms of the correlation distribution in tissue expression pattern between SAGE and microarray platforms. An ANOVA analysis was carried out that the result (P value = 0.26) indicated there is no significant differences of mean correlation coefficients between the three groups of probesets.

### The difference in tissue gene expression pattern between platforms could be partially explained by the original assignment of SAGE tags to genes, mapping of SAGE tags and array probe sets to alternative splicing variants or different regions of genes

There are many instances that more than one Affymetrix probe sets or SAGE tags are mapped to the same locus. For a given locus, there are some probe sets having tissue expression patterns both positively and negatively correlated with that of SAGE accessions [Additional file 1] at a statistically significant level (α = 0.05). Positively correlated cases were included as positive controls. We carried out an in-depth analysis of these loci in order to understand what might contribute to the discrepancies of expression profiles across various tissues measured by the two profiling platforms. A total of 33 loci [Additional file 1] were examined to understand the reasons that for the same gene locus, some SAGE tag and array probe set pairs have a positively correlated gene expression pattern while others are negatively correlated. We examined the SAGE tags and Affymetrix probe sets at the sequence level. BLAST was used to determine if SAGE tags or array probe set target sequences were indeed matched to the genes they were assigned to based on NCBI or NetAffx annotations.

**Figure 5:**
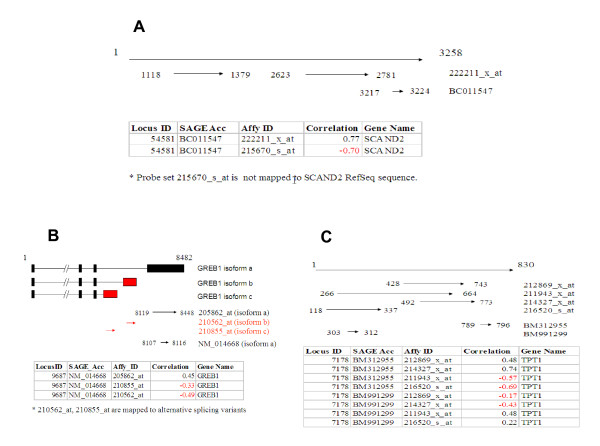
Representative illustrations of SAGE tag and probe set mapping to RefSeq sequences. (A) SAGE tags and probe sets were not mapped to the same Locus by BLAST; (B) SAGE tags and probe sets were mapped to different splicing variants; (C) SAGE tags and probe sets were mapped to different regions of the same gene.

Our analysis revealed that the lack of positive correlations in tissue expression patterns between SAGE and Affymetrix platforms for most of 33 loci in Additional file 1 can be explained by one of 3 factors: (1) Prior to statistical analysis of the data, SAGE tags were mapped to UniGene and subsequently LocusLink IDs through their GenBank accession numbers based on annotations provided by NCBI. Affymetrix array probe set IDs were mapped to LocusLink IDs according to NetAffx annotation. However, when SAGE tag and array probe set target sequences are searched against human RefSeq sequence database using BLAST, some of the tags or probe sets failed to match or only show weak similarity to a RefSeq sequence. Therefore, it is not surprising that the expression data for these SAGE tags and array probe sets are not positively correlated. For example, probe set 215670_s_at and 222211_x_at are mapped to gene SCAND2 (Locus link ID: 54581) based on NetAffx annotation and SAGE tag BC011547 is mapped to gene SCAND2 using NCBI annotation. However, when we ran BLAST to verify the mapping, we discovered that probe set 215670_s_at failed to show significant sequence similarity to SCAND2 Refseq full length sequence. The incorrect probe set mapping could be the main reason that SAGE tag BC011547 has negative correlation with probe set 215670_s_at, although it is positively related with probe set 222211_x_at (Figure 5A). The differences in tissue expression profiles of several other gene loci that we examined could also be explained by incorrect assignments of SAGE tags to genes (data not shown); (2) In several cases, SAGE tags and array probe sets are mapped to different splicing variants of the same gene locus. A representative graph is shown in Figure 5B. The original SAGE tag is represented by NM_014668 that was mapped to locus ID 9687 (Figure 5B). Three probe sets are linked to the same locus based on NetAffx annotation. The tissue expression patterns between SAGE and GDS596 contain both positive and negative correlations. Figure 5B strongly suggests that negative correlations are results of two probe sets being mapped to a different splice variant of the same gene locus. (3). SAGE tags and array probe sets are mapped to different sequence regions of the same RefSeq full-length sequence. An example is illustrated in Figure 5C. 4 array probe sets (212869_x_at, 211943_x_at, 214327_x_at and 216520_s_at) and 2 SAGE tags (BM312955 and BM991299) are mapped to the same RefSeq sequence of TPT1 gene. While tag BM312955 and probe sets 212869_x_at and 214327_x_at align to the 3' portion of the sequence, tag BM991299 and probe sets 211943_x_at and 216520_s_at are mapped toward the middle and the 5' end of the full length sequence. Correlation coefficient values between the 2 SAGE tags and 4 probe sets clearly demonstrate that SAGE tags and array probe sets mapped to the proximal locations of gene sequences have positively correlated gene expression profiles. In contrast, if SAGE tags and array probe sets map to different locations, their gene expression patterns are negatively correlated.

## Discussion

Tissue expression pattern of a gene provides an important clue to the potential roles of a gene in biological processes and its potential utility as a drug target or biomarker. In this report, we took advantage of vast amount of expression data in the public domain, and specifically examined the expression pattern of genes under normal physiological states across variety of tissues with data generated by the two most widely used technologies, SAGE and microarray. The reason that we chose to examine tissue expression patterns rather than absolute expression levels for each gene is based on several obvious reasons: (1) the tissue samples used in different studies are from different sources; (2) we wanted to avoid the systematic biases introduced by each technology in expression profiling; (3) we want to examine the feasibility and practicality of utilizing public expression data for answering tissue-specific gene expression.

Of more than one thousand SAGE tag and probe set pairs that correspond to the same gene using LocusLink-based NetAffx and NCBI annotation, the correlations in tissue expression pattern of a gene between the two technology platforms are modest, with 42–54% of genes showing significant correlations. Further analysis reveals that 30–40% of genes whose expression patterns are positively correlated and 10–15% of genes whose expression patterns are negatively correlated (Table 3). The number of pairs between SAGE tags and probe sets compared were limited by the availability of data from the public gene expression repository. One simple explanation for the modest correlation is the variations in the biological samples used in different laboratories and different technologies. However, this appeared not to be the cause, as indicated by our finding that the correlation within a tissue types from these public repository is compatible with that of same tissue samples in direct SAGE and microarray comparison studies [7]. In addition, the modest correlation is not likely caused by other spurious correlations due to small number of common tissue types between datasets generated by two technologies (Figure 2), by gene abundance across tissues (Figure 3) or by different array probe design (Figure 4), or by gene function.

Due to the fact that a full length gene can be represented by multiple probe sets and SAGE tags, it was not surprising that correlations in a gene can be both positive and negative depending upon tag and probe set pairs (Table 3 and 5), which accounts for 10% of total significant correlation pairs. When they were examined in depth through sequence analyses, some of the discrepancies can be explained by incorrect assignments of SAGE tags to full-length genes. In our initial data processing, SAGE tags were assigned to LocusLink IDs based on EST sequence mapping in NCBI annotation databases. This is also the approach employed by Lu et al. in their study. The accuracy of such an approach is compromised because when the SAGE data in our study were re-analyzed, many tags were found to be incorrectly assigned to ESTs, possibly because the EST sequence database was less comprehensive and complete as of today. Therefore, we recommend that in order to avoid misinterpretation of SAGE gene expression data, SAGE tags should be directly mapped to LocusLinks and RefSeq sequences using BLAST. Our analysis has revealed that mapping SAGE tags and array probe sets to different splice variants or different sequence regions of the same gene also contribute to the lack of positive correlations between tissue expression patterns, suggesting these factors need to be taken into consideration when one compares SAGE and array expression profiles.

Public expression data repositories such as GEO are rich resources for biologist to mine [1], and exciting discoveries are being made with these resources [3,10]. However, due to the lack of standardization across the different platforms that was used for generating the deposited data, the exploitation of this rich resource requires extensive knowledge about the technologies. This report further indicates that experimental methods and informatic methodologies for better interoperability among platforms remain to be done. Similar to previous studies [6,11], our analysis demonstrated there are significant discrepancies between the gene expression platforms. However, to our knowledge, this is the first report to de-convolute the reasons for the discrepancies. Our in-depth analysis suggests that the difference in tissue gene expression pattern between platforms could be at least partially explained by the original assignment of SAGE tags to genes, mapping of SAGE tags and array probe sets to alternative splicing variants or different regions of genes. Therefore, one of the areas to be improved is more accurate SAGE tag assignment to full-length gene. We are currently working on reassigning all public SAGE tags to the latest release of human genome assembly through BLAST sequence analysis.

## Methods

### Data source

All data were downloaded from Gene Expression Omnibus web site [12]. Specifically, SAGE datasets were from two platforms, namely GPL4 and GPL6, as part of Cancer Genome Anatomy Project (CGAP) SAGE library collection that includes data from normal and cancerous tissues. SAGE tag counts per million (TPM) were used in data analysis. Microarray data were from GDS596 (Affymetrix U133A platform), GDS594 and GDS181(Affymetrix U95Av2 platform), and these data were generated with a wide array of tissues, organs of the normal physiological state. Microarray gene expression signal values were downloaded and normalized to the same scale that 2% trimmed means were set to 500. Tissue sample annotations were downloaded and carefully examined to match corresponding microarray or SAGE data for each normal tissue (Table 1).

### Mapping of SAGE tags and array probe sets to genes

Human UniGene annotation file Hs.data was downloaded from NCBI and parsed to retrieve GenBank accession numbers of sequences that were used to generate each UniGene cluster. SAGE tags were then mapped to UniGene through their representative GenBank accession numbers and subsequently mapped to LocusLink IDs based on LocusLink annotation file loc2UG downloaded from NCBI. SAGE tags were also directly mapped to RefSeq sequences by BLAST. Affymetrix array probe set IDs were mapped to LocusLink IDs based on NetAffx annotation [13]. A total of 7536 loci were common between SAGE and array datasets. Then, subset of data was extracted from SAGE and microarray datasets with shared Locus IDs.

### Data processing

For each of SAGE or Affymetrix array platform, since there are multiple samples for each tissue, we first computed correlation coefficients of gene expression values between different samples of the same tissue type. Since correlation coefficients are greater than 0.9 for most tissues (data not shown), gene expression values are averaged from multiple samples for further analysis. In SAGE data, not every gene has tag count in all tissues due to the depth of the original tag sequencing effort (Table 2), therefore, we only kept genes with data points from at least five tissue types (of 13 total normal tissue types) for further analyses. Data were processed in Microsoft Excel, Access, Spotfire and JMP5.1.

For each gene locus, expression data from SAGE or microarray were Z transformed to have a distribution with mean = 0 and variance = 1. Then Pearson's correlation coefficients were calculated for each gene between SAGE and microarray datasets and the significance of correlation was approximated with Student's t for n-2 degrees of freedom, t=r/(1−r2)/(n−2)
 MathType@MTEF@5@5@+=feaafiart1ev1aaatCvAUfKttLearuWrP9MDH5MBPbIqV92AaeXatLxBI9gBaebbnrfifHhDYfgasaacH8akY=wiFfYdH8Gipec8Eeeu0xXdbba9frFj0=OqFfea0dXdd9vqai=hGuQ8kuc9pgc9s8qqaq=dirpe0xb9q8qiLsFr0=vr0=vr0dc8meaabaqaciaacaGaaeqabaqabeGadaaakeaacqWG0baDcqGH9aqpcqWGYbGCcqGGVaWldaGcaaqaaiabcIcaOiabigdaXiabgkHiTiabdkhaYnaaCaaaleqabaGaeGOmaidaaOGaeiykaKIaei4la8IaeiikaGIaemOBa4MaeyOeI0IaeGOmaiJaeiykaKcaleqaaaaa@3D92@[14].

## Authors' contributions

SDL, YHL, TW, BL carried out data analyses. EWS and KD gave the critical review of manuscript and support. SDL and BL conceived, designed and coordinated the study, and draft the manuscript.

## Reviewers' comments

### Reviewer's report 1

Dr. I. King Jordan, National Center for Biotechnology Information, Bethesda, MD 20894

Li et al present a comparative analysis of human gene expression data generated with two distinct high throughput experimental techniques: microarray analysis (from Affymetrix oligonucleotide chips) and serial analysis of gene expression (SAGE). The most important conclusion of this work is the substantial discordance between tissue-specific expression patterns revealed by these two techniques. The observed discrepancies are primarily attributed to problems with mapping SAGE tags to genes and, in particular, differences in the gene (transcript) locations that SAGE tags and array probes map to.

This work reports important results that should be of substantial interest, not to mention concern, to biologists working with large scale gene expression data sets. The overall analysis is succinct and clearly presented. The authors took care to try and account for the source of the discrepancies between microarray and SAGE data sets. The work could benefit from a few more analytical controls aimed at normalizing the comparison between these two very different methods. I elaborate on the issue of controls below. A bit more detail on methods would also be helpful.

1. One outstanding issue is the relative sensitivity of the two methods. Affymetrix chips have a defined lower limit for detecting gene expression, while SAGE has no such theoretical limit. SAGE should be able to detect tiny levels of gene expression given a large enough library of sequenced tags. In practice though SAGE libraries are of limited size and this dramatically affects their sensitivity. In other words, a count of 0 from a large SAGE library may have a very different meaning than a count of 0 from a small SAGE library. It has been reported that SAGE libraries of size 120,000–160,000 sequenced tags are needed to approximate the sensitivity of Affymetrix chips (Lu et al. Genomics 2004 84:631). The SAGE libraries analyzed in this report range from an order of magnitude smaller than this threshold (GSM764–13,302) to almost large enough (GSM14750–118,083). This size difference would seem to substantially compromise the ability to compare tissue-specific gene expression vectors between platforms. The authors deal with this issue tangentially by only comparing tissues where there exists a SAGE tag count (i.e. no SAGE values of 0 included). They do show that there is no relationship between the number of common tissue types and correlations between methods as well as no relationship between expression level and correlation between methods. In addition to these controls, it would be interesting to know if gene expression vectors that are drawn from libraries of similar size ranges, particularly those from larger libraries, have higher correlations between methods.

#### Author's response

This is a good point. However, there are not enough public data we can use to calculate the relationship between the size of libraries and correlations between two methods.

2. Differences in mapping microarray probe sequences to genes versus SAGE tag sequences to genes also account for differences between expression patterns measured with the two techniques. In this paper, probes and tags were first mapped to transcripts and then to genes (loci) and then gene-specific expression patterns were compared. It would be interesting to know the extent to which probe and tag sequences can be matched exactly (or at least overlap substantially) through direct comparison, and whether expression patterns revealed by probe and tag sequences that correspond in this way are more similar than what the authors observed using the gene-centric approach.

#### Author's response

We examined if the probes and tags fall into same regions of gene (Figure 5) in this paper. Our results indicated that expressions derived from array probes and tags mapped to the same regions of genes are indeed more closely correlated than those from different regions. The next logic step is to do the exact match between SAGE tag sequences and probe sequences as suggested by reviewer. However, because probe and tag sequences are relatively short, it is uncommon to have exactly matched or substantially overlapping probe and tag sequences. Furthermore, this comparison requires examining array gene expression at the probe level instead of the probesets, which poses a technical barrier because array gene expression data based on individual probes are generally unavailable. Nevertheless, we recognize that the approach suggested by the reviewer would certainly help to de-convolute the discrepancies between the two gene expression platforms.

3. There are numerous cases where different microarray probes and different SAGE tags map to the same genes. Taking these cases into consideration, it would be interesting to know if they show similar correlations between tissue specific expression patterns for within method comparisons than seen for the between method comparisons reported here.

#### Author's response

Our main purpose is to compare the tissue specific expression between microarray and SAGE. Since the correlation coefficients between different samples of same tissue type within the same method is typically higher 0.9 (in Method section).

4. Both SAGE platforms analyzed here use 10 bp tags. More recent SAGE technology works on 17 bp tags and results in better tag-to-gene mapping.

It would be interesting to know whether 17 bp SAGE platforms yield better correlations with Affymetrix microarray data. Is there enough data from 17 bp libraries to test this?

#### Author's response

We have not been able to locate the enough data from 17 bp libraries.

5. It is not clear from the methods whether the authors use SAGE tag counts or SAGE tag counts per million (TPM). This point is critical since the latter method allows for count comparisons between libraries. It would also be helpful to explicate which values were used for the Affymetrix data.

#### Author's response

It is TPM for SAGE. For microarray, we downloaded gene expression signal values and normalized different datasets to the same scale. This information was added to the method section (Methods, paragraph 1).

6. It is difficult to rigorously visually evaluate whether there is any trend between expression level and correlation between methods from Figure 3. It might help to provide a trend line or at least some quantitative indication of no relationship.

#### Author's response

Figure 3 has been remade.

### Reviewer's report 2

Dr Joel Bader, Johns Hopkins University

1. This manuscript provides a comparison of tissue-specific gene expression inferred from microarray data and from serial analysis of gene expression (SAGE) sequencing-based methods.

There has been much previous work comparing these methods. The most pertinent paper I found, which also considers tissue profiles, is "Huminiecki, Lloyd, and Wolfe, 'Congruence of tissue expression profiles from Gene Expression Atlas, SAGEmap, and TissueInfo databases', BMC Genomics 2003, 4: 31". These authors characterized concordance using R-squared measures of transcript enrichment as reported by different databases (rather than technical assessments by running an identical biological sample across several platforms). They found that R-squared ranged from 0.1 to 0.9 depending on the comparison and on the tissue.

Others have compared identical samples run using SAGE and Affy chips, for example Ishii et al., 'Direct comparison of GeneChip and SAGE on the quantitative accuracy in transcript profiling analysis', Genomics 2000, 68: 136. These authors find that agreement is better for more highly-expressed transcripts. Occasionally, they find strong disagreement between SAGE and chip results. They propose several reasons: (1) Strong hybridization to the mismatch probe may yield a spurious inference of under-expression. (2) Probes from the 5' end of the gene may be less effective. (3) Annotations of gene structure may be incorrect, leading to the wrong sequence on the chip. (4) SAGE tags may be ambiguous, and alternative splicing may make it difficult to identify the source gene.

The authors cite work on technical comparisons. Their aim is more in line with the previous work of Huminiecki et al., to evaluate the feasibility of combining public database information derived from different expression technologies.

Their results are similar to previous work. Some of their results seem imprecisely stated. For example, Results describing Fig. 1, 'There was similar number of negative correlation as positive correlation, with slightly more positive correlated genes between the platforms.' My view of Fig. 1 is that there is a highly significant enrichment of positive correlation, even though the effect size may not be as large as one would hope. The data processing methods should be more explicit about mathematical transformation. For example, I expect that log-transforms were used and that transformation to a unit normal was done by subtracting the mean and dividing by the standard deviation.

#### Author's response

The wording was changed (Page 6, last paragraph). Both SAGE data and microarray data were Z-transformed to have a distribution with mean = 0 and variance = 1. Description of data transformation was provided in the method section (Methods, paragraph 3).

2. One interesting result reported by the authors is that the class of Affy probe (_at, _s_at, _x_at) does not seem to affect the results. It would be an improvement to devise a test statistic to make this statement more quantitative. Could the authors perform ANOVA using the probe class as an explanatory variable for the correlation between SAGE and microarray?

#### Author's response

We followed reviewers' advice and carried out an ANOVA analysis that confirmed our statement the class of Affy probe (_at, _s_at, _x_at) does not seem to affect the results (P value = 0.26). We further performed pairwise t-tests with multiple testing adjustment and the results are consistent with the ANOVA analysis. These additional analyses have been added to the result section (Results, paragraph 9).

3. The factors leading to disagreement between Affy and SAGE data largely agree with what previous publications have suggested: improper mappings from SAGE tags or Affy probes to genes; differential expression of splice variants that are measured by one method but not the other; and mapping to different sequence regions.

#### Author's response

Previous publications have only suggested the similar factors leading to the disagreement between Affy and SAGE data, however, no data and detailed analyses were conducted before.

4. The authors suggest that remapping the SAGE tags to an updated annotation of human genes would increase the utility of the data. This is a good idea. It would be interesting to see whether remapping changes any of the conclusions. I suspect that agreement between SAGE and array data will be improved, but the effect will be small.

#### Author's response

We agree with reviewers' assessment. We are planning to do, however, due to the time and computing power required for this question, we will share in a different venue in the future.

5. The manuscript would be improved by highlighting results or conclusions that differ from previous joint analysis of this type of data. The weakest point of the manuscript is that it is not clear whether anything new has been learned. The language usage should be improved.

#### Author's response

We have taken the suggestion. Statements have been added to the abstract (Abstract, paragraph 3) and the discussion (Discussion, 1^st ^and last paragraph) sections to highlight the novelty in our work.

### Reviewer's report 3

Dr Arcady Mushegian, Stowers Institute, Kansas City

1. The authors use some simple and straightforward exploratory statistics to ask whether gene expression data from different platforms can be directly compared. They conclude that, as a general rule, the answer is no: though many tissue-specific gene expression values are positively correlated between two platforms, i.e., SAGE and Affymetrix oligonucleotide arrays, many others are not.

The authors list several reasons why this could be so, but pursue and provide evidence for only one hypothesis, namely that there are errors in assigning SAGE tags or Affy oligos to genes.

It is a useful note of caution to those who attempt to 'integrate' expression data, analogous to an advice to infer function and evolutionary relationships of genes/proteins from their sequences and not the keywords in the FASTA definition lines. The need to know which genes are being examined is a necessarily quality control step that has to precede any gene expression analysis. If mistakes are found, they have to be corrected in order to make biological inferences. Unfortunately, this work does not provide much more than some support to this quite self-evident statement. In my opinion, the article is neither a novel scientific observation nor even a technical advance.

#### Author's response

We are glad the reviewer pointed out that a careful examination of gene identity should always precede integration of gene expression data, as suggested by our report. This notion might be self-evident to experienced bioinformatics scientists but not necessarily to most bench scientists. Therefore, we believe it is of great importance to make scientific community aware of our analysis.

From reviewers' comment, we also recognize that one area for improvement in our manuscript is to emphasize the novelty of our work. For example, we provided evidence not only for the hypothesis that there are errors in assigning SAGE tags or Affy oligos to genes, but also for other reasons we listed to explain the discrepancies between the two gene expression platforms. In Figure 5, we show that mapping probesets or SGAE tags to different splice variants or different regions of the same gene could be the cause of differential gene expression patterns between microarray and SAGE. These results have not been reported by previous studies. We added several statements in the abstract (Abstract, paragraph 3) and the discussion (Discussion, 1^st ^and last paragraph) sections to highlight the scientific contributions of our work.

2. On the other hand, I would be extremely curious to know whether there are bona fide genes that consistently show poor cross-platform correlation of expression levels, and whether this, together with the knowledge of biases of Affymetrix and SAGE, could be used to infer something interesting about gene regulation.

#### Author's response

We have found that genes showing poor cross-platform correlation of expression levels did not fall into any particular functional class (Results, paragraph 7, Additional file 1).

## Supplementary Material

Additional file 1 Both positive and negative correlation within a given locus. The data contains the names and descriptions of gene loci with both positive and negative correlations between SAGE tag accession and Affymetrix probe ID. Click here for file

## References

[B1] Barrett T, Suzek TO, Troup DB, Wilhite SE, Ngau WC, Ledoux P, Rudnev D, Lash AE, Fujibuchi W, Edgar R (2005). NCBI GEO: mining millions of expression profiles--database and tools. Nucleic Acids Res.

[B2] Ventura B (2005). Mandatory submission of microarray data to public repositories: how is is working. Physiol Genomics.

[B3] Mootha VK, Lepage P, Miller K, Bunkenborg J, Reich M, Hjerrild M, Delmonte T, Villeneuve A, Sladek R, Xu F, Mitchell GA, Morin C, Mann M, Hudson TJ, Robinson B, Rioux JD, Lander ES (2003). Identification of a gene causing human cytochrome c oxidase deficiency by integrative genomics. Proc Natl Acad Sci U S A.

[B4] Lockhart DJ, Dong H, Byrne MC, Follettie MT, Gallo MV, Chee MS, Mittmann M, Wang C, Kobayashi M, Horton H, Brown EL (1996). Expression monitoring by hybridization to high-density oligonucleotide arrays. Nat Biotechnol.

[B5] Velculescu VE, Zhang L, Vogelstein B, Kinzler KW (1995). Serial analysis of gene expression. Science.

[B6] Ishii M, Hashimoto S, Tsutsumi S, Wada Y, Matsushima K, Kodama T, Aburatani H (2000). Direct comparison of GeneChip and SAGE on the quantitative accuracy in transcript profiling analysis. Genomics.

[B7] Lu J, Lal A, Merriman B, Nelson S, Riggins G (2004). A comparison of gene expression profiles produced by SAGE, long SAGE, and oligonucleotide chips. Genomics.

[B8] van Ruissen F, Ruijter JM, Schaaf GJ, Asgharnegad L, Zwijnenburg DA, Kool M, Baas F (2005). Evaluation of the similarity of gene expression data estimated with SAGE and Affymetrix GeneChips. BMC Genomics.

[B9] (2006). http://www.affymetrix.com/technology/design/index.affx.

[B10] Mootha VK, Bunkenborg J, Olsen JV, Hjerrild M, Wisniewski JR, Stahl E, Bolouri MS, Ray HN, Sihag S, Kamal M, Patterson N, Lander ES, Mann M (2003). Integrated analysis of protein composition, tissue diversity, and gene regulation in mouse mitochondria. Cell.

[B11] Huminiecki L, Lloyd AT, Wolfe KH (2003). Congruence of tissue expression profiles from Gene Expression Atlas, SAGEmap and TissueInfo databases. BMC Genomics.

[B12] (2006). http://www.ncbi.nlm.nih.gov/projects/geo/.

[B13] (2006). http://www.affymetrix.com/analysis/index.affx.

[B14] Dawson B, Trapp R, Dawson B and Trapp RS (2001). Research questions about relationships between variables. Basic &Clinical Biostatistics.

