# Supplementary material for: Too much data, but little inter-changeability: a lesson learned from mining public data on tissue specificity of gene expression
Source: Biol Direct. 2006 Oct 25;1:33. doi: 10.1186/1745-6150-1-33 (PMC1634740; doi:10.1186/1745-6150-1-33)
Supplement: Additional file 1 — Both positive and negative correlation within a given locus. The data contains the names and descriptions of gene loci with both positive and negative correlations between SAGE tag accession and Affymetrix probe ID. [file 1745-6150-1-33-S1.doc]

**Table 5. Both positive and negative correlation within a given locus**

| **LocusID** | **SAGE_Acc** | **Affy_ID** | **Pos_Corr** | **SAGE_Acc** | **Affy_ID** | **Neg_Corr** | **GeneName** | **Description** |
| --- | --- | --- | --- | --- | --- | --- | --- | --- |
| 163 | NM_001282 | 200612_s_at | 0.71 | AI538783 | 200615_s_at | -0.27 | AP2B1 | adaptor-related protein complex 2, beta 1 subunit |
| 960 | BC004372 | 217523_at | 0.50 | BC004372 | 204489_s_at | -0.51 | CD44 | CD44 antigen (homing function and Indian blood group system) |
| 3069 | BC001179 | 221767_x_at | 0.17 | T55446 | 221767_x_at | -0.47 | HDLBP | high density lipoprotein binding protein (vigilin) |
| 3191 | NM_001533 | 221860_at | 0.66 | NM_001533 | 202072_at | -0.78 | HNRPL | heterogeneous nuclear ribonucleoprotein L |
| 3192 | BC015782 | 200593_s_at | 0.65 | AW377040 | 200593_s_at | -0.57 | HNRPU | heterogeneous nuclear ribonucleoprotein U (scaffold attachment factor A) |
| 3192 | AW377040 | 216855_s_at | 0.62 | AW377040 | 200593_s_at | -0.57 | HNRPU | heterogeneous nuclear ribonucleoprotein U (scaffold attachment factor A) |
| 3192 | BC015782 | 200594_x_at | 0.95 | AW377040 | 200593_s_at | -0.57 | HNRPU | heterogeneous nuclear ribonucleoprotein U (scaffold attachment factor A) |
| 3192 | BC015782 | 200593_s_at | 0.65 | AW377040 | 216868_s_at | -0.80 | HNRPU | heterogeneous nuclear ribonucleoprotein U (scaffold attachment factor A) |
| 3192 | AW377040 | 216855_s_at | 0.62 | AW377040 | 216868_s_at | -0.80 | HNRPU | heterogeneous nuclear ribonucleoprotein U (scaffold attachment factor A) |
| 3192 | BC015782 | 200594_x_at | 0.95 | AW377040 | 216868_s_at | -0.80 | HNRPU | heterogeneous nuclear ribonucleoprotein U (scaffold attachment factor A) |
| 3309 | AI491789 | 211936_at | 0.91 | BC020235 | 211936_at | -0.64 | HSPA5 | heat shock 70kDa protein 5 (glucose-regulated protein, 78kDa) |
| 3309 | X87949 | 211936_at | 0.82 | BC020235 | 211936_at | -0.64 | HSPA5 | heat shock 70kDa protein 5 (glucose-regulated protein, 78kDa) |
| 3953 | BE615001 | 207255_at | 0.53 | BE615001 | 202377_at | -0.52 | LEPR | leptin receptor |
| 3953 | BE615001 | 211354_s_at | 0.76 | BE615001 | 202377_at | -0.52 | LEPR | leptin receptor |
| 3953 | BE615001 | 211356_x_at | 0.49 | BE615001 | 202377_at | -0.52 | LEPR | leptin receptor |
| 5257 | W16838 | 202739_s_at | 0.58 | NM_000293 | 202739_s_at | -0.41 | PHKB | phosphorylase kinase, beta |
| 5257 | W16838 | 202738_s_at | 0.67 | NM_000293 | 202739_s_at | -0.41 | PHKB | phosphorylase kinase, beta |
| 5688 | BC004427 | 216088_s_at | 0.80 | BM562909 | 216088_s_at | -0.63 | PSMA7 | proteasome (prosome, macropain) subunit, alpha type, 7 |
| 5688 | BC004427 | 201114_x_at | 0.94 | BM562909 | 216088_s_at | -0.63 | PSMA7 | proteasome (prosome, macropain) subunit, alpha type, 7 |
| 5862 | AF070629 | 208730_x_at | 0.79 | AF070629 | 208734_x_at | -0.69 | RAB2 | RAB2, member RAS oncogene family |
| 6157 | BM997745 | 203034_s_at | 1.00 | BC005326 | 212044_s_at | -0.87 | RPL27A | ribosomal protein L27a |
| 6157 | BM997745 | 216421_at | 0.70 | BC005326 | 212044_s_at | -0.87 | RPL27A | ribosomal protein L27a |
| 6157 | BC005326 | 203034_s_at | 0.82 | BC005326 | 212044_s_at | -0.87 | RPL27A | ribosomal protein L27a |
| 6157 | BM997745 | 203034_s_at | 1.00 | BM997745 | 212044_s_at | -0.90 | RPL27A | ribosomal protein L27a |
| 6157 | BM997745 | 216421_at | 0.70 | BM997745 | 212044_s_at | -0.90 | RPL27A | ribosomal protein L27a |
| 6157 | BC005326 | 203034_s_at | 0.82 | BM997745 | 212044_s_at | -0.90 | RPL27A | ribosomal protein L27a |
| 6181 | BC005354 | 200909_s_at | 0.74 | BC005354 | 200908_s_at | -0.80 | RPLP2 | ribosomal protein, large P2 |
| 6421 | BM702823 | 214016_s_at | 0.49 | BM702823 | 201586_s_at | -0.52 | SFPQ | splicing factor proline/glutamine rich (polypyrimidine tract binding protein associated) |
| 6421 | BM702823 | 201585_s_at | 0.35 | BM702823 | 201586_s_at | -0.52 | SFPQ | splicing factor proline/glutamine rich (polypyrimidine tract binding protein associated) |
| 6421 | BM702823 | 221768_at | 0.42 | BM702823 | 201586_s_at | -0.52 | SFPQ | splicing factor proline/glutamine rich (polypyrimidine tract binding protein associated) |
| 6711 | BF346832 | 214856_at | 0.79 | BG289924 | 213914_s_at | -0.81 | SPTBN1 | spectrin, beta, non-erythrocytic 1 |
| 6711 | BG289924 | 214856_at | 0.66 | BG289924 | 213914_s_at | -0.81 | SPTBN1 | spectrin, beta, non-erythrocytic 1 |
| 6731 | AI423215 | 211454_x_at | 0.84 | AI423215 | 208803_s_at | -0.75 | SRP72 | signal recognition particle 72kDa |
| 6731 | AI423215 | 208263_at | 0.89 | AI423215 | 208803_s_at | -0.75 | SRP72 | signal recognition particle 72kDa |
| 6731 | AI423215 | 211454_x_at | 0.84 | AI423215 | 208095_s_at | -0.82 | SRP72 | signal recognition particle 72kDa |
| 6731 | AI423215 | 208263_at | 0.89 | AI423215 | 208095_s_at | -0.82 | SRP72 | signal recognition particle 72kDa |
| 6867 | NM_006283 | 200911_s_at | 0.64 | NM_006283 | 217433_at | -0.64 | TACC1 | transforming, acidic coiled-coil containing protein 1 |
| 7178 | BM312955 | 214327_x_at | 0.74 | BM312955 | 216520_s_at | -0.69 | TPT1 | tumor protein, translationally-controlled 1 |
| 7178 | BC022436 | 212869_x_at | 0.93 | BM312955 | 216520_s_at | -0.69 | TPT1 | tumor protein, translationally-controlled 1 |
| 7178 | BM991299 | 211943_x_at | 0.48 | BM312955 | 216520_s_at | -0.69 | TPT1 | tumor protein, translationally-controlled 1 |
| 7178 | BM312955 | 214327_x_at | 0.74 | BC022436 | 211943_x_at | -0.67 | TPT1 | tumor protein, translationally-controlled 1 |
| 7178 | BC022436 | 212869_x_at | 0.93 | BC022436 | 211943_x_at | -0.67 | TPT1 | tumor protein, translationally-controlled 1 |
| 7178 | BM991299 | 211943_x_at | 0.48 | BC022436 | 211943_x_at | -0.67 | TPT1 | tumor protein, translationally-controlled 1 |
| 7846 | BC006468 | 209118_s_at | 0.86 | K00557 | 209118_s_at | -0.64 |  | tubulin, alpha 3 |
| 8570 | NM_003685 | 204371_s_at | 0.73 | NM_003685 | 212303_x_at | -0.91 | KHSRP | KH-type splicing regulatory protein (FUSE binding protein 2) |
| 8727 | NM_003798 | 202468_s_at | 0.48 | NM_003798 | 213712_at | -0.35 | CTNNAL1 | catenin (cadherin-associated protein), alpha-like 1 |
| 8795 | NM_147187 | 209294_x_at | 0.76 | NM_147187 | 209295_at | -0.50 | TNFRSF10B | tumor necrosis factor receptor superfamily, member 10b |
| 8795 | NM_147187 | 209294_x_at | 0.76 | NM_147187 | 210405_x_at | -0.64 | TNFRSF10B | tumor necrosis factor receptor superfamily, member 10b |
| 9470 | BC005392 | 213570_at | 0.71 | BC005392 | 213571_s_at | -0.89 | EIF4E2 | eukaryotic translation initiation factor 4E member 2 |
| 9556 | AF116639 | 210532_s_at | 0.35 | AF116639 | 202279_at | -0.24 | C14orf2 | chromosome 14 open reading frame 2 |
| 9637 | NM_005102 | 210704_at | 0.43 | NM_005102 | 202305_s_at | -0.24 | FEZ2 | fasciculation and elongation protein zeta 2 (zygin II) |
| 9659 | AB042555 | 212390_at | 0.63 | AB042555 | 214129_at | -0.88 | PDE4DIP | phosphodiesterase 4D interacting protein (myomegalin) |
| 9659 | AB042555 | 212390_at | 0.63 | AB042555 | 212392_s_at | -0.71 | PDE4DIP | phosphodiesterase 4D interacting protein (myomegalin) |
| 9659 | AB042555 | 212390_at | 0.63 | AB042555 | 215575_at | -0.73 | PDE4DIP | phosphodiesterase 4D interacting protein (myomegalin) |
| 9687 | NM_014668 | 205862_at | 0.45 | NM_014668 | 210855_at | -0.33 |  | GREB1 protein |
| 9687 | NM_014668 | 205862_at | 0.45 | NM_014668 | 210562_at | -0.49 |  | GREB1 protein |
| 10159 | BC010395 | 201442_s_at | 0.82 | BC010395 | 201444_s_at | -0.64 | ATP6AP2 | ATPase, H+ transporting, lysosomal accessory protein 2 |
| 10209 | BC005118 | 212225_at | 0.66 | BE936952 | 212225_at | -0.61 |  | putative translation initiation factor |
| 10209 | BC005118 | 202021_x_at | 0.94 | BE936952 | 212225_at | -0.61 |  | putative translation initiation factor |
| 10209 | BC005118 | 212227_x_at | 0.98 | BE936952 | 212225_at | -0.61 |  | putative translation initiation factor |
| 10209 | BC005118 | 212130_x_at | 0.97 | BE936952 | 212225_at | -0.61 |  | putative translation initiation factor |
| 10552 | NM_006409 | 209309_at | 0.24 | NM_006409 | 200950_at | -0.23 | ARPC1A | actin related protein 2/3 complex, subunit 1A, 41kDa |
| 10728 | BC003005 | 200627_at | 0.21 | BE829650 | 200627_at | -0.55 |  | unactive progesterone receptor, 23 kD |
| 10791 | BC017891 | 204929_s_at | 0.97 | BC001634 | 214115_at | -0.85 | VAMP5 | vesicle-associated membrane protein 5 (myobrevin) |
| 11079 | AJ001421 | 202297_s_at | 0.71 | AJ001421 | 213114_at | -0.73 |  | RER1 homolog (S. cerevisiae) |
| 25941 | BC015178 | 212055_at | 0.58 | BC015178 | 213616_at | -0.28 | C18orf10 | chromosome 18 open reading frame 10 |
| 54581 | BC011547 | 222211_x_at | 0.77 | BC011547 | 215670_s_at | -0.70 | SCAND2 | SCAN domain containing 2 |
